# MetaDisorder: a meta-server for the prediction of intrinsic disorder in proteins

**DOI:** 10.1186/1471-2105-13-111

**Published:** 2012-05-24

**Authors:** Lukasz P Kozlowski, Janusz M Bujnicki

**Affiliations:** 1 Laboratory of Bioinformatics and Protein Engineering, International Institute of Molecular and Cell Biology, ul, Trojdena 4, 02-109, Warsaw, Poland; 2 Laboratory of Bioinformatics, Institute of Molecular Biology and Biotechnology, Faculty of Biology, ul Umultowska 89, 61-614, Poznan, Poland

## Abstract

**Background:**

Intrinsically unstructured proteins (IUPs) lack a well-defined three-dimensional structure. Some of them may assume a locally stable structure under specific conditions, e.g. upon interaction with another molecule, while others function in a permanently unstructured state. The discovery of IUPs challenged the traditional protein structure paradigm, which stated that a specific well-defined structure defines the function of the protein. As of December 2011, approximately 60 methods for computational prediction of protein disorder from sequence have been made publicly available. They are based on different approaches, such as utilizing evolutionary information, energy functions, and various statistical and machine learning methods.

**Results:**

Given the diversity of existing intrinsic disorder prediction methods, we decided to test whether it is possible to combine them into a more accurate meta-prediction method. We developed a method based on arbitrarily chosen 13 disorder predictors, in which the final consensus was weighted by the accuracy of the methods. We have also developed a disorder predictor GSmetaDisorder3D that used no third-party disorder predictors, but alignments to known protein structures, reported by the protein fold-recognition methods, to infer the potentially structured and unstructured regions. Following the success of our disorder predictors in the CASP8 benchmark, we combined them into a meta-meta predictor called GSmetaDisorderMD, which was the top scoring method in the subsequent CASP9 benchmark.

**Conclusions:**

A series of disorder predictors described in this article is available as a MetaDisorder web server at http://iimcb.genesilico.pl/metadisorder/. Results are presented both in an easily interpretable, interactive mode and in a simple text format suitable for machine processing.

## Background

Many proteins are functional despite they lack a stable three-dimensional structure under physiological conditions in vitro and/or in vivo [1,2]. Regions of protein-protein and protein-nucleic acid interactions, as well as sites of posttranslational modification, often fall into regions that are locally disordered or undergo disorder–order transition in biologically relevant situations [3,4]. Intrinsic disorder is a common feature of “hub” proteins that interact with multiple other proteins and perform important regulatory roles in the cell [5]. Many intrinsically unstructured proteins (IUPs) or intrinsically unstructured regions (IURs) are critical for cell survival, proliferation, differentiation, and apoptosis, which make them important from a biomedical point of view.

Intrinsically unfolded proteins, once purified, can be identified by various experimental methods [6-9]. However, experimental determination of the absence of a three-dimensional structure is difficult. Since the presence or the absence of a single stable structure is encoded in the protein sequence, it is possible to use the sequence information to predict regions of disorder in the similar manner as e.g. secondary structure. Therefore, the emerging “unfoldomics” field [1,10] has prompted the development of numerous computational methods for the prediction of disordered regions from protein sequence (see e.g. list of URLs in DisProt, the Database of Protein Disorder [11]).

IUPs and intrinsically unfolded regions (IURs) are quite diverse. They can be classified in various ways according to length (short vs long disorder), method of experimental determination (e.g. “lack of electron of density in crystal structures”), the presence or absence of certain structural features (e.g. disorder with secondary structure but no tertiary structure), and many other factors. Different types of disorder are often associated with different types of characteristic. For this reason, some computational methods for disorder predictions are available in several versions, trained on different datasets, e.g. on short and long IURs separately [1,2]. However, thus far no single clear-cut classification of all disorder types has emerged that would be accepted and used by all experts in the field, and most methods for disorder prediction from protein sequence aim for a binary classification of protein residues: ordered or disordered (i.e. will all types of disorder treated as a single class).

The so-called “meta-method” approach relies on the fact that different algorithms have their individual advantages and disadvantages, and the combination of methods can be used to improve the prediction accuracy. This approach has been used to develop many successful prediction methods, e.g. in protein fold recognition [12], protein function prediction [13], prediction of protein domains [14], prediction of protein model quality [15], and recently also in protein disorder prediction [16-18]. In this article, we describe a set of predictors that take as an input a protein sequence, query other methods, and calculate a final “consensus” prediction of disorder (in the sense of “any disorder” as a single class, as opposed to different types of order treated jointly as another single class). They have been implemented as a single web server called MetaDisorder, available at http://iimcb.genesilico.pl/metadisorder/. One of our methods is essentially a primary predictor, as it does not use any other disorder prediction method, however it is “meta” in the sense that it does utilize other predictions, namely alignments to proteins of known structure reported by protein fold-recognition methods. Our other disorder predictors are typical meta-methods, as they directly query a series of primary disorder predictors and utilize their output. Additionally, other types of one-dimensional features, such as predicted secondary structure and predicted solvent accessibility are used. In the framework of the CASP8 and CASP9 benchmarks, these meta-predictors outperformed other methods for disorder prediction [19].

## Methods

### Definition of disorder

Protein disorder can be defined by many ways depending on the research focus and experimental method used. As a baseline, we used the definition used in the Critical Assessment of protein Structure Prediction (CASP) experiments: the disordered residues are those marked by REMARK465 tag in the experimentally determined protein structures deposited in Protein Data Bank (PDB) [20], which indicates regions with missing coordinates in crystal structures determined by X-ray crystallography or residues with highly variable coordinates in ensembles of Nuclear Magnetic Resonance (NMR) structures. This definition was extended to include also proteins deposited in the DisProt database (disorder validated experimentally by a variety of experimental methods such as circular dichroism (CD) spectroscopy, mass spectrometry, immunochemistry, SDS-PAGE gel, small-angle X-ray scattering (SAXS), currently over 1300 regions) [11]. The advantage of the DisProt database is that it includes proteins without known three-dimensional structure, especially proteins that are entirely disordered, whose structure typically cannot be determined by high resolution methods (X-ray crystallography and NMR). Thus, we treat all disorder types as a single class.

### Primary methods used in the meta-method

The MetaDisorder series of predictors combined, via a machine-learning approach, the predictions of 13 primary disorder predictors that performed well in CASP and are freely available as standalone applications or stable web servers that can process large numbers of queries: DisEMBL [21], DISOPRED2 [22], DISpro [23], Globplot [24], iPDA [25], IUPred [26], Pdisorder [27], Poodle-s [28], Poodle-l [29], PrDOS [30], Spritz [31], DisPSSMP [32], and RONN [33]. Additionally, the meta-predictors designed for CASP9 used also six subjectively selected methods for protein fold-recognition: HHSEARCH run over PDB70 and CDD databases [34], FFAS [35], mGenThreader [36], PSI-BLAST run in two different modes (with and without masking regions with low sequence complexity) over the culled PDB database [37], PHYRE [38], and PCONS [39] (a consensus method that uses as an input models generated by MODELLER [40] based on alignments from the previously mentioned fold-recognition methods). For short description of each method see Table 1 and Table 2. Additionally, two methods for secondary structure prediction: JNET [41] and PSIPRED [42], and one solvent accessibility predictor, JNET [41], were used.

**Table 1:** Description of disorder predictors analyzed in this work

**Method**	**Short description**	**Availability**	**Ref.**
DisEMBL	ANN trained to predict classic loops (DSSP), flexible loops with high B-factors, missing coordinates in X-ray structures, regions of low-complexity and prone to aggregation.	local installation	[21]
DISOPRED2	SVM trained to predict residues with missing coordinates.	local installation	[22]
DISpro	Recursive neural networks (RNNs) trained to predict missing coordinates.	local installation	[23]
GlobPlot	A simple method based on several hydrophobicity scales to predict regions of missing coordinates and loops with high B-factors.	local installation	[24]
iPDA	Incorporates information about sequence conservation, predicted secondary structure, sequence complexity and hydrophobic clusters.	web service	[25]
IUPred	Estimates pairwise interaction energies using a statistical potential. Two versions for predicting long and short disorder.	web service	[26]
Pdisorder	Combination of neural network, linear discriminant function and acute smoothing procedure is used for recognition of disordered and ordered regions in proteins.	web service	[27]
Poodle-s	SVM trained for short disorder detection (uses PSSMs generated by PSI-BLAST).	web service	[28]
Poodle-l	Predicts long disorder using an SVM.	web service	[29]
PrDOS	Predicts missing coordinates in 3D structure using SVM and PSSMs from PSI-BLAST.	web service	[30]
Spritz	Predicts long and short disorder (missing coordinates) using two separate SVMs. Utilizes secondary structure.	web service	[31]
RONN	Predicts missing coordinates using an ANN.	local installation	[33]

**Table 2:** Description of fold recognition methods used by MetaDisorder

**Method**	**Short description**	**Availability**	**Ref.**
PSI-BLAST	Position-Specific Iterated BLAST uses position-specific scoring matrices derived during the search of the nr database	local installation	[37]
FFAS	Profile-profile alignment and fold-recognition algorithm for fold and function assignment	local installation	[35]
mGenThreader	The method combines profile-profile alignments with secondary-structure specific gap-penalties, classic pair- and solvation potentials using a linear combination optimized with a regression SVM model	local installation	[36]
HHsearch	Generalizes the alignment of protein sequences with a profile hidden Markov model (HMM) to the case of pairwise alignment of profile HMMs	local installation	[34]
PCONS	A neural-network-based consensus predictor	local installation	[39]
PHYRE	An algorithm that uses profile-profile and secondary structure matching algorithm	web service	[38]

### Training and testing datasets

To train the meta-predictors, two independent datasets were used. The first dataset was prepared based on the combined DisProt database (version 3.6) and CASP7 targets. Sequences longer than 1000 residues were omitted, because they exceed the length limit of some of the primary methods used and could not be processed automatically without arbitrary manipulations. Overall, this procedure provided 566 proteins, which included 232,664 residues in total, of which 23.45% were disordered. The second dataset, called pdbRemark465, was based on structures in the PDB database. Representative structures were extracted using the PISCES server [43] and filtered according to the following criteria: experimental technique: X-ray crystallography, resolution < 2 Å, R-factor < 0.2, length 50–1000 aa residues, and mutual sequence similarity < 20%. The resulting dataset contained 1147 proteins (289,008 residues, of which 6.28% were disordered according to the REMARK465 tag in the PDB files, see Additional file 1). In the final version of the meta-predictor, we combined these two datasets and used them for assessing the disorder prediction accuracy. During that procedure, standard 10-fold cross validation was used. All amino acid residues were randomly assigned into 10 bins of nearly equal size. 9 bins were used as a source of the training data and the remaining 10th bin was used as a source of the testing data. This procedure was then repeated 10 times, with each of the 10 bins used exactly once for validation. The results of 10 analyses were then averaged to produce final scores.

Since we aimed to be as objective as possible in assessing the predictive power of our methods in a fair comparison to other methods, to avoid any bias we tested all predictors described in this article within truly blind tests of CASP8 and CASP9, in which (as mentioned earlier), the prediction of disorder is defined as the ability to identify regions with missing coordinates in crystal structures determined by X-ray crystallography or residues with highly variable coordinates in ensembles of NMR structures.

For the training of GSmetaDisorder3D and GSmetaDisorderMD predictors, we used proteins from CASP8 (122 proteins, 27,614 residues, of which 11.11% were disordered; among them 19 were solved by NMR, 2.515 residues, of which 47.95% were disordered). Again, 10-fold cross validation was used. The detailed statistics about each dataset are provided in Table 3.

**Table 3:** Summary of the datasets employed in this study

	**DisProt + CASP7**	**pdbRemark465**	**CASP8**
Number of proteins	566	1147	122
Number of residues in disordered regions	54,570 (23.45%)	18,146 (6.28%)	3,068 (11.11%)
Number of residues in ordered regions	178,094 (76.55%)	270,862 (93.72%)	24,546 (88.89%)
Total number of residues	232,664	289,008	27,614

### Measures used for training and evaluation

The results of predictions can be divided into four categories: true positives (TP) – residues correctly predicted as disordered, true negatives (TN) – residues correctly predicted as ordered, false positives (FP) – ordered residues misclassified as disordered, and false negatives (FN) – disordered residues misclassified as ordered.

The first assessment criterion we used was the receiver operating characteristic (ROC). The ROC curve is a graphical plot of the sensitivity vs. false positive rate for a classifier, as its discrimination threshold is changed. The resulting area under curve (AUC) defines the overall robustness of an algorithm, where 1 means the perfect predictor (all true positives are found by the method without any false positives) and 0.5 corresponds to a random one.

The second criterion is the weighted score, called S_w,_ which rewards a correct disorder prediction higher than a correct order prediction [44]. This is done to avoid over-prediction of an ordered state due the fact that ordered regions are more common in known proteins. The S_w_ score is defined as:$S_{W}=\frac{S}{S_{\max}}=\frac{W_{\mathrm{disorder}}TP-W_{\mathrm{order}}FP+W_{\mathrm{order}}TN-W_{\mathrm{disorder}}FN}{W_{\mathrm{disorder}}(TN+FN)+W_{\mathrm{order}}(TN+FP)}$

where the W_disorder_ equals the fraction of ordered residues and W_order_ equals the fraction of disordered residues. S_w_ is in the range −1 to 1, where 0 means random prediction. Maximization of S_w_ was the main criterion of the optimization procedure and it was also used to assess the relative value of individual primary disorder predictors to be incorporated into our meta-servers. The S_w_ score was directly used as a weight of a prediction returned by each such method.

The third commonly used measure, which was not used during our procedure of developing the consensus methods, but which was used for their evaluation, is Matthews correlation coefficient (MCC) [45]:$MCC=\frac{TP\cdot TN-FP\cdot FN}{\sqrt{\left(TP+FP)(TP+FN)(TN+FP)(TN+FN\right)}}$S_w_ and MCC were the measures used during CASP to assess disorder predictors.

Finally, we used our own measure, called S_ww,_ which combines AUC and S_w_ score in the following way: it is calculated using the S_w_ formula, but the discrimination threshold is changed incrementally from 0 to 1, by steps of 0.01, giving sets of TP, TN, FP, FN values that are used to calculate a series of S_w_ scores. S_ww_ is the average value of these scores. This score was used only in the GSmetaDisorderMD2 method during CASP9.

The statistical significance of the evaluation scores was determined by the bootstrap confidence interval method [19,46]: 80% of the targets were randomly selected 1000 times, and the mean absolute error of scores was calculated. The ROC statistics were compared by using the Wilcoxon signed rank test and by calculating standard errors of ROC statistics.

### Binary consensus and continuous consensus versions of MetaDisorder predictors

In general, two categories of predictors exist. The simplest predictors are binary, they try to classify the predicted feature only into separate subcategories (here disordered and ordered residues). More advanced methods return continuous scores with values e.g. between 0 and 1 that inform how certain the prediction is, and the prediction is made according to an arbitrarily chosen threshold. The lower the threshold, the higher the number of both true and false positives. Accordingly, initially we constructed two versions of the MetaDisorder predictor, named BinCons and FloatCons. These two methods were tested within the framework of the CASP8 benchmark as groups with numbers 153 and 297, respectively [19]. BinCons uses only binary predictions from primary methods: each disorder prediction for a residue is counted as 1 and ordered as 0.01 (0 was avoided to prevent possible cases of dividing by zero). FloatCons uses all the information available: if a given method returns a continuous prediction, its score is used during the final consensus calculation. A consensus score for each residue is calculated by summing the scores from all primary methods and multiplying them by the accuracy of the given method. The result is normalized, i.e. the score is divided by the maximal possible score. For simplicity, the criterion of a method’s accuracy used as the weight of the method was S_w_ calculated for our combined datasets. It was possible, because S_w_ does not depend on the predictor output type.

In the next step, a special correcting function is used. It takes into account the fact that residues located in the protein termini are on the average more disordered than residues in the middle of the protein chain. This function is based on the statistics of disorder presence in the 15 proximal residues calculated on both datasets and provides an appropriate corrective factor, by which the original predictive score is multiplied.

Finally, the decision whether a residue is ordered or disordered is made. If a residue scores above the threshold, it is predicted as disordered; otherwise it is predicted as ordered. The threshold for classifying the residue as ordered or disordered was based on S_w_ scores obtained during 10-fold cross validation tests.

Additionally, at the end, the repairing procedure is employed to improve the prediction. For predicted string (e.g. “DDD‒‒‒D‒‒…”, with D indicating disorder and “-” indicating order) a simple smoothing filter with a window of five residues is applied. It eliminates short (up to 3 residues) stretches of predicted disorder within long regions of predicted order (converts previous example to “DDD‒‒‒‒‒‒…”).

### GSmetaDisorder3D – a template-matching method

Apart from disorder predictors, many other bioinformatics tools yield implicit or explicit information about order and disorder. In the course of a variety of other protein sequence analysis projects, we realized that there is a clear correlation between the disorder in the target protein sequence, and the presence of gaps in alignments to structurally characterized templates calculated by the protein fold-recognition methods. Although the implementation of a method utilizing this type of information may seem trivial, it was not so straightforward to deal with different types of fold recognition methods. In other words, it was not so obvious which method should be used or, if many methods were used, how to rank them. Additionally, a template-matching method should be able to take into account the fact that matches to homologous proteins have different reliability and in some cases homologous sequences cannot be found. To address all these questions, we compared the results from arbitrary chosen fold recognition methods that were relatively fast and performed well in the framework of CASP: HHSEARCH, FFAS, mGenThreader, PSI-BLAST, PHYRE, and PCONS5 (see Methods for details and references). To optimize the weights assigned to individual methods depending on the alignment quality we used a genetic algorithm implemented in Pyevolve [47]. The fitness function of the genetic algorithm was designed as a one-dimensional vector of length 24 (8 methods mentioned above multiplied by 3 thresholds for well-, moderately- and poorly-scored templates; see Table 4 for details of the thresholds used). In this way, the weights for all methods were obtained, for the further incorporation into a combined template-matching method. The resulting predictor was tested in CASP9 as a group number 421 (GSmetaDisorder3D).

**Table 4:** Thresholds used in fold recognition programs for classification of potentially good, medium and poor alignments

	**Predicted alignment quality**		
Method	Good	Medium	Poor
PSI-BLAST*	< 2e-06	< 0.023	> 0.023
FFAS	<−34.5	< −8.5	> − 8.5
MGenThreader	> 0.65	> 0.546	< 0.546
HHsearch*	>95	>80	<80
PCONS	> 2.17	> 1.03	< 1.03
PHYRE	< 0.085	< 0.27	> 0.27

* - the same score was used regardless of the database.

### GSmetaDisorderMD and GSmetaDisorderMD2 – combined disorder consensus and template-matching method

The next method in the MetaDisorder series, GSmetaDisorderMD, was developed by combining FloatCons (the consensus method with continuous scoring) with GSmetaDisorder3D (the method based on analysis of gaps in fold-recognition alignments). The same genetic algorithm was used as in the training of GSmetaDisorder3D, but additionally the second dimension to the vector was added to optimize the relationship between these two components. This method was tested in CASP9 as a group number 374.

GSmetaDisorderMD2 is a variant of GSmetaDisorderMD, in which the genetic algorithm used for training optimized the S_ww_ score instead of the S_w_ score. This predictor was tested in CASP9 as a group number 147.

### Implementation and availability

**Figure 1:**
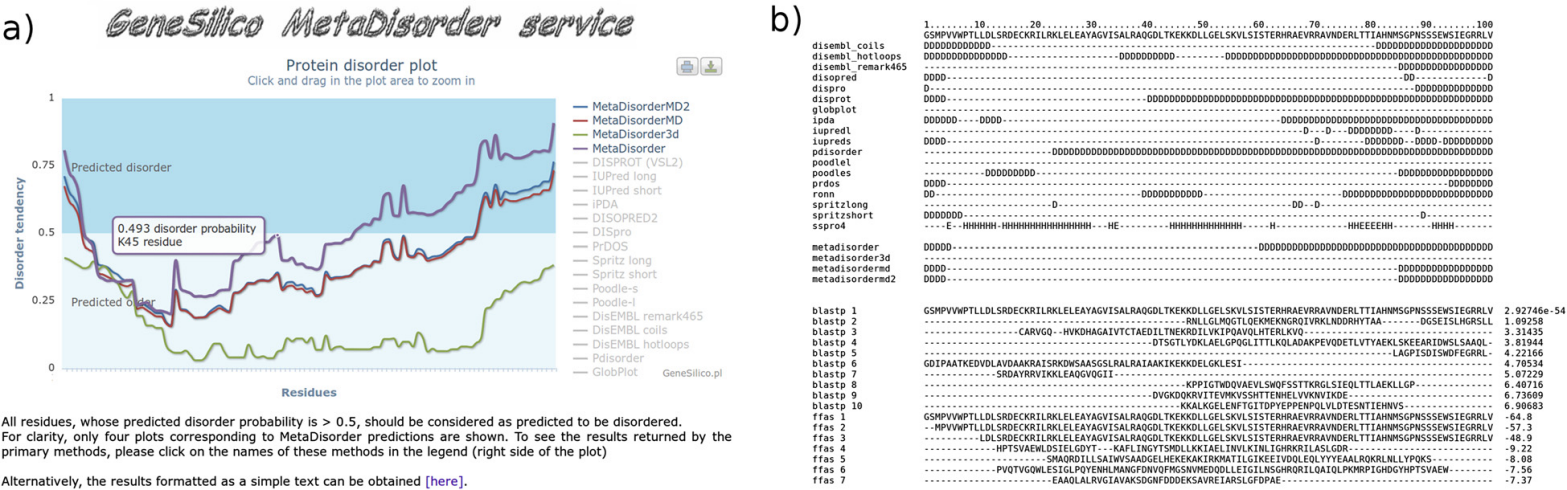
**MetaDisorder web-server interface.a)** user-friendly web interface – main plot part can be easily zoomed in and out, results reported by all primary methods can be downloaded in the CASP format. **b)** simple text output format suitable for machine processing.

The MetaDisorder is a web interface to our series of disorder meta-predictors and can be accessed at http://iimcb.genesilico.pl/metadisorder/. Wrappers and parsers for primary prediction methods were written in the Python programming language under the Unix system. Data are stored in a MySQL database. The web server was implemented using the mod_python Apache module. For the interactive presentation of results, the JavaScript chart library Highcharts [48] is used. Additionally, the results of analyses can be also obtained as simple text output (for details see Figure 1).

## Results

### Meta prediction of protein disorder from primary disorder predictors

**Figure 2:**
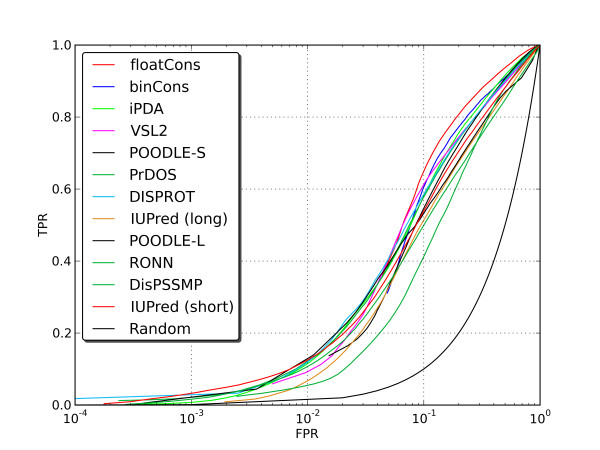
**Receiver operating characteristics (ROC) plots and their area under curve (AUC) for disorder prediction methods used to construct the FloatCons meta-predictor for a combined dataset comprising Disprot, CASP7 targets and PDBremark465.** FPR values are presented on a logarithmic scale.

Motivated by the success of meta-prediction in various fields of bioinformatics, we tested its applicability to the prediction of disordered residues in protein sequences. Initially, we developed meta-predictors BinCons and FloatCons that calculate a consensus score by taking into account the relative expected accuracies of constituent primary methods (see Methods for details). BinCons and FloatCons were first benchmarked by ourselves on combined datasets consisting of CASP7 targets, DISPROT database and pdbRemark465 dataset obtained from a filtered PDB database (Table 5 and Figure 2, see Methods for details) and subsequently by independent assessors within the framework of the CASP8 experiment (Table 6) [19]. In both tests the BinCons and FloatCons meta-predictors performed considerably better than individual primary predictors (e.g. AUC of 0.868 and 0.843 compared to 0.830 and 0.829 for the top-performing primary predictors iPDA and VSL2 in our benchmark). The statistical significance of those results was compared by using the Wilcoxon signed rank test (for details see Additional file 2: Table S1). The overall difference of accuracy between these two meta-predictors was relatively small (2.9%), but statistically significant according to the Wilcoxon signed rank test. The difference between both meta-predictors and iPDA and VSL2 is also statistically significant. This exercise demonstrated that meta-prediction can significantly improve the inference of intrinsic disorder from protein sequence, but the use of continuous scores contributes little to that success over simple binary prediction.

**Table 5:** Performance of disorder prediction on the combined pdbRemark465, CASP7 and Disprot dataset

	**Evaluation score**		
Method	Sw	MCC	AUC
FloatCons	**0.608 ± 0.007**	0.475 **±** 0.008	**0.868 ± 0.002**
BinCons	0.599 **±** 0.007	**0.487 ± 0.008**	0.843 ± 0.003
iPDA	0.555 **±** 0.006	0.419 **±** 0.006	0.829 ± 0.004
DISPROT(vls2)	0.539 **±** 0.005	0.399 **±** 0.005	0.830 ± 0.001
DISOPRED	0.481 **±** 0.006	0.436 **±** 0.006	0.778 ± 0.003
POODLE-S	0.474 **±** 0.009	0.423 **±** 0.010	0.828 ± 0.004
PrDOS	0.469 **±** 0.007	0.442 **±** 0.008	0.810 ± 0.006
POODLE-L	0.464 **±** 0.010	0.397 **±** 0.010	0.794 ± 0.004
RONN	0.450 **±** 0.006	0.350 **±** 0.007	0.762 ± 0.006
IUPred (short)	0.445 **±** 0.006	0.412 **±** 0.007	0.788 ± 0.002
DisPSSMP	0.442 **±** 0.012	0.377 **±** 0.012	0.776 ± 0.004
IUPred (long)	0.432 **±** 0.008	0.392 **±** 0.009	0.787 ± 0.004
Spritz (long)	0.418 **±** 0.009	0.377 **±** 0.010	-
Pdisorder	0.383 **±** 0.007	0.350 **±** 0.007	-
Dispro	0.355 **±** 0.006	0.411 **±** 0.008	-
Spritz (short)	0.334 **±** 0.007	0.306 **±** 0.007	-
DisEMBL	0.289 **±** 0.007	0.232 **±** 0.006	-
GlobPlot	0.187 **±** 0.004	0.172 **±** 0.004	-

The highest value for each score is shown in bold.

**Table 6:** The results of our meta-predictors and top-scoring primary methods in CASP8 and CASP9

**CASP8**				
Method	Sw	AUC	Sensitivity	Specificity
FloatCons	**0.662 ± 0.048**	0.908 ± 0.017	**0.758 ± 0.048**	0.904 ± 0.004
BinCons	0.661 **±** 0.050	0.897 ± 0.021	0.741 ± 0.050	0.920 ± 0.003
DisoClust	0.644 **±** 0.047	0.908 ± 0.018	0.727 ±0.047	0.917 ± 0.004
MULTICOM	0.660 ± 0.039	0.896 ± 0.019	0.796 ± 0.039	0.864 ± 0.004
Mahmood-Torda	0.619 ± 0.061	**0.918 ± 0.015**	0.641 ± 0.061	**0.978 ± 0.001**
POODLE-L	0.588 ± 0.066	0.895 **±** 0.021	0.646 ± 0.066	0.942 ± 0.004
**CASP9**				
Method	Sw	AUC	Sensitivity	Specificity
FloatCons	0.427 ± 0.009	0.795 ± 0.011	0.574 ± 0.020	0.854 ± 0.009
GSmetaDisorder3D	0.391 ± 0.007	0.784 ± 0.012	0.411 ± 0.016	**0.948 ± 0.008**
GSmetaDisorderMD	0.476 ± 0.006	0.818 ± 0.008	**0.654 ± 0.012**	0.821 ± 0.010
GSmetaDisorderMD2	**0.516 ± 0.010**	0.841 ± 0.014	**0.653 ± 0.013**	0.860 ± 0.012
PrDOS2	0.509 ± 0.002	**0.855 ± 0.010**	0.609 ± 0.008	0.857 ± 0.003
MULTICOM-REFINE	0.500 ± 0.003	0.821 ± 0.008	0.651 ± 0.003	0.851 ± 0.004

The highest value for each score is shown in bold.

### Gaps in fold recognition alignments provide useful information for protein disorder prediction

Subsequently, we have developed a primary disorder predictor GSmetaDisorder3D that uses information from the coverage of the target sequence by known protein structures, according to alignments reported by protein-fold recognition methods (hence, it is “primary” with respect to disorder prediction, but “meta” with respect to utilization of other predictors). These methods aim at aligning target protein sequences to proteins with related structure. The lack of matches to known structures for a given sequence region may indicate the lack of detectable structured counterparts in the database, including cases of structural disorder. Figure 1b illustrates an example, where the paucity of matches to known structures reported by fold-recognition methods corresponds to a disordered region. GSmetaDisorder3D uses six different protein fold-recognition methods (with two of these run in two different modes). The selection of these tools was dictated by the methods’ accuracy (according to CASP [49]), but also speed, and either availability for local installation or stability of a web service. One issue we had to address was the fact that each fold-recognition method typically generates up to ten alternative alignments that are scored differently and may exhibit different accuracy. There are many nonlinear aspects of these methods that should be taken into account when considering the prediction of disorder using information from homologous alignments. To address them, we employed a genetic algorithm. The fitness function was designed in such a way that it optimizes a vector of size 24, where triads of the vector elements represents weights for the eight fold recognition methods indicating good, medium and poor quality alignments.

As it can be seen in Table 6, GSmetaDisorder3D performs better than many primary disorder prediction methods that sometimes use sophisticated machine learning algorithms, although it does not outperform them all. According to our benchmark, this method achieved ROC of 0.833 on CASP8 targets (Table 7). This indicates that the coverage of the target sequence by known structures in fold-recognition alignments is a good discriminator of protein order and disorder, but alone it is not sufficient to predict protein disorder as well as the top disorder prediction methods.

**Table 7:** The results of evaluation of GSmetaDisorder3d, GSmetaDisorderMD and GSmetaDisorderMD2 on CASP8 targets

	**Evaluation score**		
Method	MCC	Sw	AUC
floatCons	**0.654 ± 0.041**	0.606 **±** 0.023	0.904 **±** 0.009
GSmetaDisorder3d	0.589 **±** 0.047	0.519 ± 0.024	0.833 **±** 0.014
GSmetaDisorderMD	0.558 ± 0.034	**0.684 ± 0.023**	0.927 **±** 0.011
GSmetaDisorderMD2	0.607 ± 0.042	**0.684 ± 0.022**	**0.929 ± 0.017**

### Fold-recognition analysis adds value to consensus disorder prediction

The GSmetaDisorder3D was not intended to serve as an independent predictor, but as a complement to other methods based on different principles. It has been combined with the consensus meta-predictor FloatCons into a meta-predictor named GSmetaDisorderMD. According to an in-house benchmark and CASP9, GSmetaDisorderMD outperforms FloatCons by 2-4%, depending on the dataset used for testing (see Table 6 and Table 7 for numeric details). It must be emphasized that this method was tested only on CASP targets (with ten cross validation across residues), because only for them predictions from all primary methods were available.

We have also developed and tested a minor variant of this method, dubbed GSmetaDisorderMD2, trained with the use of the S_ww_ score instead of the S_w_ score as the target function. This modification brought about a small but significant improvement in the prediction quality, especially if we consider the results from CASP9 (AUC = 0.841 and 0.818 for GSmetaDisorderMD2 and GSmetaDisorderMD, respectively).

## Discussion

### Consensus predictions are practically useful: they are significantly better than primary predictors

The development of meta-predictors is often criticized as a parasitic approach that discourages the development of primary methods and does not improve our understanding of the underlying biological processes. In this article we have described not only a series of meta-methods that use other developers’ methods, but a novel primary method based on a different principle, which does not “beat” other primary algorithms in a head-to-head comparison, but is sufficiently different, that its inclusion improves meta-prediction by a few percent. Thus, we argue that the development of meta-servers can actually positively influence the development of methods that are based on novel principles and that it can highlight the utility of new algorithms even if they do not “win” the competition on the basic level. On the other hand, our benchmarks demonstrate that many “old” methods are still useful in terms of contribution of important information that can be used for meta-prediction, and that meta-predictors can incorporate them as “building blocks” into a practically useful bioinformatics service.

The key conclusion from our work is that even a very simple weighted consensus (binCons and floatCons predictors) is able to improve disorder prediction over primary methods, resulting in a more robust and accurate prediction, as assessed according both to the S_w_ score and AUC. As can be concluded from data presented in Table 5 and Table 6, regardless of the type of score and dataset used, consensus methods performed comparatively well both in our in-house benchmark and in CASP [19]. The most advanced and best-performing meta-predictors described in this manuscript use machine learning to derive the best features from the primary predictors available. They outperformed consensus predictors based on simply averaging the input of the primary predictors.

### Consensus predictions improve other methods’ predictions. Where does the improvement come from?

Consensus predictors are more robust than primary predictors they are based on. They give less false positives and on the average the predictions are more definite. Primary predictors are different from each other and in a collective prediction their different strengths can be combined and/or their difference weaknesses can be eliminated. First, different datasets are used for training, biasing the prediction towards (or against) certain types of proteins with particular features. For instance, the use of proteins from the PDB eliminates all proteins that are so disordered that their structure cannot be determined, while the use of proteins from DisProt implies the reliance on low-resolution experimental data that blurs the boundary between order and disorder. Second, different machine learning techniques are used that can be more or less accurate under different circumstances. Typically, the impact of the machine learning algorithm used or the parameters chosen for the training of a given predictor is not clear, as comprehensive evaluation of various machine-learning methods with respect to a particular dataset is rarely performed and described. Hence, each primary predictor can be viewed as an instantiation of its developers’ expertise and ideas with respect to the dataset preparation, invention of new algorithms and/or machine learning use, which is never fully optimal with respect to all relevant parameters. A successful meta-predictor based on a machine-learning approach is able to perform a synthesis of abilities of the primary methods, and in our opinion the greatest improvement comes from eliminating their individual deficiencies rather than in the exploitation of the individual unusual strengths.

### Deficiencies of the meta-server approach for disorder prediction

Disorder predictors developed in this work were carefully benchmarked against many other methods, using several different datasets as a reference, including the blind tests of CASP8 and CASP9, where they always ranked among top contenders. It is unfortunately impossible to compare these methods to all the published disorder predictors (as of December 2011, over 60 methods can be found in the literature and on the web), as not all of them are freely available as servers or standalone tools, and not all of them participate in CASP.

Another problem in benchmarking bioinformatics methods is that almost all of them use as an initial step a similarity search over some protein sequence database (usually with the PSI-BLAST [37] method). These databases are constantly updated. For this reason it is not entirely fair to compare our predictors with other methods, unless they are installed locally and use the same databases. Hence, we could not directly compare our method to many new methods. For example the MFDp meta-predictor [50] can be installed locally, but it depends on more than ten third-party programs (e.g. HHsearch [34]), which use their own databases. A fair comparison of MFDp and MetaDisorder methods would require e.g. the availability of HHsearch HMM-profile databases from 2008 and 2010 and others, which are unfortunately not available.

The problem with local benchmarks mentioned above emphasizes the importance of CASP experiments. There, the contenders cannot control the dataset used for testing the methods, and the problem with biological database content is alleviated, as all methods are allowed to use the most up-to-date sequence databases (whether they actually use the full potential of the availability of these databases is another question). Hence, it should be stressed out that the presented series of methods was developed, tested, and improved through two editions of CASP, and was found to be superior to other methods in these fair competitions.

MetaDisorder is relatively slow, as it depends on more than 20 programs, which are not very fast even if installed locally. Some of them search big databases and/or are not parallelized. For instance the generation of alignments by fold recognition methods can take more than an hour for long sequences. In the case of online web servers installed on third-party servers, the response may be delayed for reasons that are beyond the control of the meta-predictor (e.g. server crash). A significantly speed-limiting factor in our GSmetaDisorder3D method is the use of the PCONS5 algorithm, which is a fold-recognition meta-predictor run only when all primary fold recognition methods return their alignments and corresponding 3D models are generated by MODELLER. Despite these performance drawbacks, the MetaDisorder web server is typically able to calculate final predictions from within minutes up to few hours, depending on sequence length.

Probably the most serious problem in disorder prediction is that the binary classification of residues into the ordered or disordered state is very simplistic. “Disorder” is not a single state, but in fact represents a whole range of biophysical characteristics that can be captured by different experimental techniques. It has been shown that disorder predictors trained on proteins with different types of disorder often achieve poor accuracy on disorder of proteins of a different type, which has led to the definition of “flavors” of disorder, characterized by differences in sequence properties [51]. There are certain classes of disorder for which specialized predictors have been developed, for instance short vs. long disorder [28,29], and prediction of protein-binding regions in disordered proteins [52]. The use of a meta-server allows not only for combining predictions of different flavors of disorder into one “consensus” prediction, but also to collect and display these different predictions next to each other, allowing the human user to make an informed functional interpretation. On the other hand, the collection of results obtained by multiple methods can be overwhelming for a lay user. Clearly, there is a need to develop more clear-cut classification of disorder that would capture functional features correlated with sequence features that can be used by machine learning methods in the development of multi-state disorder predictors. Current efforts towards the development of disorder ontology (http://www.disprot.org/idpo.obo) and new classification schemes (e.g. by the ch-cdf plot method [53]) are expected to help in the development of multi-class predictors.

## Conclusions

The meta-approach allows the consolidation of pre-existing knowledge to obtain more robust and accurate predictions than with the use of primary predictors. We developed one primary disorder meta-predictor and a series of disorder meta-predictors that use different sets of primary predictors, and tested their performance on different datasets. The most important evaluation of the predictors’ accuracy was in blind tests of CASP8 and CASP9. In both cases, our meta-predictors were found to be superior with respect to all primary methods and other meta-predictors. Currently, our MetaDisorder web service offers a possibility to run more than 20 bioinformatics tools (including primary disorder predictors, secondary structure predictors, and fold recognition methods), and to analyze the summary of results via a user-friendly interface.

## Competing interests

Authors declare that they have no competing interests.

## Authors’ contributions

LPK collected all data, carried out calculations, developed programs and web interface and drafted the manuscript. JMB conceived of the project and edited the manuscript. Both authors read and approved the final manuscript.

## Supplementary Material

Additional file 1 1147 sequences with their definitions of being disordered/ordered extracted from pdb files according to remark465. Click here for file

Additional file 2 **Table S1.** Results of the Wilcoxon Singed-Rank Two-Sided Tests for the AUC scores on dataset combining CASP7, DISPROT and pdbRemark465 datasets. Click here for file

## References

[B1] Dunker AK Oldfield CJ Meng J Romero P Yang JY Chen JW Vacic V Obradovic Z Uversky VN The unfoldomics decade: an update on intrinsically disordered proteins BMC Genomics 2008 9 Suppl 2 S1 10.1186/1471-2164-9-S2-S1 PMC2559873 18831774

[B2] Tompa P Fuxreiter M Fuzzy complexes: polymorphism and structural disorder in protein-protein interactions Trends Biochem Sci 2008 33 1 2 8 10.1016/j.tibs.2007.10.003 18054235

[B3] Zhang Y Stec B Godzik A Between order and disorder in protein structures: analysis of "dual personality" fragments in proteins Structure 2007 15 9 1141 1147 10.1016/j.str.2007.07.012 17850753 PMC2084070

[B4] Fuxreiter M Tompa P Simon I Local structural disorder imparts plasticity on linear motifs Bioinformatics 2007 23 8 950 956 10.1093/bioinformatics/btm035 17387114

[B5] Haynes C Oldfield CJ Ji F Klitgord N Cusick ME Radivojac P Uversky VN Vidal M Iakoucheva LM Intrinsic disorder is a common feature of hub proteins from four eukaryotic interactomes PLoS Comput Biol 2006 2 8 e100 10.1371/journal.pcbi.0020100 16884331 PMC1526461

[B6] Bernado P Mylonas E Petoukhov MV Blackledge M Svergun DI Structural characterization of flexible proteins using small-angle X-ray scattering J Am Chem Soc 2007 129 17 5656 5664 10.1021/ja069124n 17411046

[B7] Ferreon AC Moran CR Gambin Y Deniz AA Single-molecule fluorescence studies of intrinsically disordered proteins Methods Enzymol 2010 472 179 204 20580965 10.1016/S0076-6879(10)72010-3

[B8] Meier S Blackledge M Grzesiek S Conformational distributions of unfolded polypeptides from novel NMR techniques J Chem Phys 2008 128 5 052204 10.1063/1.2838167 18266409

[B9] Receveur-Brechot V Bourhis JM Uversky VN Canard B Longhi S Assessing protein disorder and induced folding Proteins 2006 62 1 24 45 16287116 10.1002/prot.20750

[B10] Uversky VN The mysterious unfoldome: structureless, underappreciated, yet vital part of any given proteome J Biomed Biotechnol 2010 2010 568068 20011072 10.1155/2010/568068 PMC2789583

[B11] Sickmeier M Hamilton JA LeGall T Vacic V Cortese MS Tantos A Szabo B Tompa P Chen J Uversky VN DisProt: the Database of Disordered Proteins Nucleic Acids Res 2007 35 Database issue D786 793 17145717 10.1093/nar/gkl893 PMC1751543

[B12] Kurowski MA Bujnicki JM GeneSilico protein structure prediction meta-server Nucleic Acids Res 2003 31 13 3305 3307 10.1093/nar/gkg557 12824313 PMC168964

[B13] Friedberg I Harder T Godzik A JAFA: a protein function annotation meta-server Nucleic Acids Res 2006 34 Web Server issue W379 381 16845030 10.1093/nar/gkl045 PMC1538919

[B14] Saini HK Fischer D Meta-DP: domain prediction meta-server Bioinformatics 2005 21 12 2917 2920 10.1093/bioinformatics/bti445 15840708

[B15] Pawlowski M Gajda MJ Matlak R Bujnicki JM MetaMQAP: a meta-server for the quality assessment of protein models BMC Bioinformatics 2008 9 1 403 10.1186/1471-2105-9-403 18823532 PMC2573893

[B16] Schlessinger A Punta M Yachdav G Kajan L Rost B Improved disorder prediction by combination of orthogonal approaches PLoS One 2009 4 2 e4433 10.1371/journal.pone.0004433 19209228 PMC2635965

[B17] Ishida T Kinoshita K Prediction of disordered regions in proteins based on the meta approach Bioinformatics 2008 24 11 1344 1348 10.1093/bioinformatics/btn195 18426805

[B18] Xue B Dunbrack RL Williams RW Dunker AK Uversky VN PONDR-FIT: a meta-predictor of intrinsically disordered amino acids Biochim Biophys Acta 2010 1804 4 996 1010 10.1016/j.bbapap.2010.01.011 20100603 PMC2882806

[B19] Noivirt-Brik O Prilusky J Sussman JL Assessment of disorder predictions in CASP8 Proteins 2009 77 Suppl 9 210 216 19774619 10.1002/prot.22586

[B20] Berman HM Bhat TN Bourne PE Feng Z Gilliland G Weissig H Westbrook J The Protein Data Bank and the challenge of structural genomics Nat Struct Biol 2000 7 Suppl 957 959 11103999 10.1038/80734

[B21] Linding R Jensen LJ Diella F Bork P Gibson TJ Russell RB Protein disorder prediction: implications for structural proteomics Structure 2003 11 11 1453 1459 10.1016/j.str.2003.10.002 14604535

[B22] Ward JJ McGuffin LJ Bryson K Buxton BF Jones DT The DISOPRED server for the prediction of protein disorder Bioinformatics 2004 20 13 2138 2139 10.1093/bioinformatics/bth195 15044227

[B23] Medina MW Gao F Naidoo D Rudel LL Temel RE McDaniel AL Marshall SM Krauss RM Coordinately regulated alternative splicing of genes involved in cholesterol biosynthesis and uptake PLoS ONE 2011 6 4 e19420 10.1371/journal.pone.0019420 21559365 PMC3084847

[B24] Linding R Russell RB Neduva V Gibson TJ GlobPlot: Exploring protein sequences for globularity and disorder Nucleic Acids Res 2003 31 13 3701 3708 10.1093/nar/gkg519 12824398 PMC169197

[B25] Su CT Chen CY Hsu CM iPDA integrated protein disorder analyzer Nucleic Acids Res 2007 35 Web Server issue W465 472 17553839 10.1093/nar/gkm353 PMC1933224

[B26] Dosztanyi Z Csizmok V Tompa P Simon I IUPred: web server for the prediction of intrinsically unstructured regions of proteins based on estimated energy content Bioinformatics 2005 21 16 3433 3434 10.1093/bioinformatics/bti541 15955779

[B27] SoftBerry - PDISORDER [http://linux1.softberry.com/berry.phtml?topic=pdisorder&group=programs&subgroup=propt]

[B28] Shimizu K Hirose S Noguchi T POODLE-S: web application for predicting protein disorder by using physicochemical features and reduced amino acid set of a position-specific scoring matrix Bioinformatics 2007 23 17 2337 2338 10.1093/bioinformatics/btm330 17599940

[B29] Hirose S Shimizu K Kanai S Kuroda Y Noguchi T POODLE-L: a two-level SVM prediction system for reliably predicting long disordered regions Bioinformatics 2007 23 16 2046 2053 10.1093/bioinformatics/btm302 17545177

[B30] Ishida T Kinoshita K PrDOS: prediction of disordered protein regions from amino acid sequence Nucleic Acids Res 2007 35 Web Server issue W460 464 17567614 10.1093/nar/gkm363 PMC1933209

[B31] Vullo A Bortolami O Pollastri G Tosatto SC Spritz: a server for the prediction of intrinsically disordered regions in protein sequences using kernel machines Nucleic Acids Res 2006 34 Web Server issue W164 168 16844983 10.1093/nar/gkl166 PMC1538873

[B32] Su CT Chen CY Ou YY Protein disorder prediction by condensed PSSM considering propensity for order or disorder BMC Bioinformatics 2006 7 319 10.1186/1471-2105-7-319 16796745 PMC1526762

[B33] Yang ZR Thomson R McNeil P Esnouf RM RONN: the bio-basis function neural network technique applied to the detection of natively disordered regions in proteins Bioinformatics 2005 21 16 3369 3376 10.1093/bioinformatics/bti534 15947016

[B34] Soding J Protein homology detection by HMM-HMM comparison Bioinformatics 2005 21 7 951 960 10.1093/bioinformatics/bti125 15531603

[B35] Jaroszewski L Rychlewski L Li Z Li W Godzik A FFAS03: a server for profile--profile sequence alignments Nucleic Acids Res 2005 33 Web Server issue W284 288 15980471 10.1093/nar/gki418 PMC1160179

[B36] Alber F Dokudovskaya S Veenhoff LM Zhang W Kipper J Devos D Suprapto A Karni-Schmidt O Williams R Chait BT The molecular architecture of the nuclear pore complex Nature 2007 450 7170 695 701 10.1038/nature06405 18046406

[B37] Altschul SF Madden TL Schaffer AA Zhang J Zhang Z Miller W Lipman DJ Gapped BLAST and PSI-BLAST: a new generation of protein database search programs Nucleic Acids Res 1997 25 17 3389 3402 10.1093/nar/25.17.3389 9254694 PMC146917

[B38] Lareau LF Inada M Green RE Wengrod JC Brenner SE Unproductive splicing of SR genes associated with highly conserved and ultraconserved DNA elements Nature 2007 446 7138 926 929 10.1038/nature05676 17361132

[B39] Wallner B Elofsson A Pcons5: combining consensus, structural evaluation and fold recognition scores Bioinformatics 2005 21 23 4248 4254 10.1093/bioinformatics/bti702 16204344

[B40] Sali A Potterton L Yuan F van Vlijmen H Karplus M Evaluation of comparative protein modeling by MODELLER Proteins 1995 23 3 318 326 10.1002/prot.340230306 8710825

[B41] Cuff JA Barton GJ Application of multiple sequence alignment profiles to improve protein secondary structure prediction Proteins 2000 40 3 502 511 10.1002/1097-0134(20000815)40:3<502::AID-PROT170>3.0.CO;2-Q 10861942

[B42] McGuffin LJ Bryson K Jones DT The PSIPRED protein structure prediction server Bioinformatics 2000 16 4 404 405 10.1093/bioinformatics/16.4.404 10869041

[B43] Wang G Dunbrack RL PISCES: recent improvements to a PDB sequence culling server Nucleic Acids Res 2005 33 Web Server issue W94 98 15980589 10.1093/nar/gki402 PMC1160163

[B44] Jin Y Dunbrack RL Assessment of disorder predictions in CASP6 Proteins 2005 61 Suppl 7 167 175 16187359 10.1002/prot.20734

[B45] Matthews BW Comparison of the predicted and observed secondary structure of T4 phage lysozyme Biochim Biophys Acta 1975 405 2 442 451 10.1016/0005-2795(75)90109-9 1180967

[B46] Carpenter J Bithell J Bootstrap confidence intervals: when, which, what? A practical guide for medical statisticians Stat Med 2000 19 9 1141 1164 10.1002/(SICI)1097-0258(20000515)19:9<1141::AID-SIM479>3.0.CO;2-F 10797513

[B47] Butterfield A Vedagiri V Lang E Lawrence C Wakefield MJ Isaev A Huttley GA PyEvolve: a toolkit for statistical modelling of molecular evolution BMC Bioinformatics 2004 5 1 10.1186/1471-2105-5-1 14706121 PMC317364

[B48] HighCharts JS [http://www.highcharts.com/]

[B49] Cozzetto D Kryshtafovych A Fidelis K Moult J Rost B Tramontano A Evaluation of template-based models in CASP8 with standard measures Proteins 2009 77 Suppl 9 18 28 19731382 10.1002/prot.22561 PMC4589151

[B50] Mizianty MJ Stach W Chen K Kedarisetti KD Disfani FM Kurgan L Improved sequence-based prediction of disordered regions with multilayer fusion of multiple information sources Bioinformatics 2010 26 18 i489 496 10.1093/bioinformatics/btq373 20823312 PMC2935446

[B51] Vucetic S Brown CJ Dunker AK Obradovic Z Flavors of protein disorder Proteins 2003 52 4 573 584 10.1002/prot.10437 12910457

[B52] Dosztanyi Z Meszaros B Simon I ANCHOR: web server for predicting protein binding regions in disordered proteins Bioinformatics 2009 25 20 2745 2746 10.1093/bioinformatics/btp518 19717576 PMC2759549

[B53] Huang F Oldfield C Meng J Hsu WL Xue B Uversky VN Romero P Dunker AK Subclassifying disordered proteins by the ch-cdf plot method Pac Symp Biocomput 2012 17 128 139 22174269

