# Supplementary material for: MetaDisorder: a meta-server for the prediction of intrinsic disorder in proteins
Source: BMC Bioinformatics. 2012 May 24;13:111. doi: 10.1186/1471-2105-13-111 (PMC3465245; doi:10.1186/1471-2105-13-111)
Supplement: Additional file 2 — Table S1. Results of the Wilcoxon Singed-Rank Two-Sided Tests for the AUC scores on dataset combining CASP7, DISPROT and pdbRemark465 datasets. [file 1471-2105-13-111-S2.doc]

**Table S1. Results of the Wilcoxon Singed-Rank Two-Sided Tests for the AUC scores on dataset combining CASP7, DISPROT and pdbRemark465 datasets**

|  | float  Cons | bin  Cons | VSL2 | Dis  PSSMP | iPDA | IUPred  short | PrDOS | DISO  PRED | IUPred long | POODLE-L | RONN | POODLE-S |
| --- | --- | --- | --- | --- | --- | --- | --- | --- | --- | --- | --- | --- |
| floatCons  binCons  VSL2  DisPSSMP  iPDA  IUPred short  PrDOS  DISOPRED  IUPred long  POODLE-L  RONN  POODLE-S | x  0.005  0.005  0.005  0.005  0.005  0.005  0.005  0.005  0.005  0.005  0.005 | x  0.007  0.005  0.005  0.005  0.005  0.005  0.005  0.005  0.005  0.005 | x  0.005  **0.241**  0.005  0.022  0.005  0.005  0.005  0.005  0.005 | x  0.007  **0.093**  0.017  **0.445**  **0.203**  **0.721**  **0.169**  0.005 | x  0.005  0.005  0.005  0.005  0.005  0.005  0.005 | x  0.005  **0.028**  **0.878**  0.009  0.005  0.005 | x  0.005  0.005  0.005  0.005  0.005 | x  **0.114**  **0.386**  **0.028**  0.005 | x  0.013  0.005  0.005 | x  0.007  0.005 | x  0.005 | x |

Bolded values highlight pairs of methods that are statistically indistinguishable from each other at the 0.05 significance level based on p-values
